# Personal

**Published:** 1898-07

**Authors:** 


### PERSONAL.

Dr. Joseph Price, of Philadelphia, visited Buffalo, on Tuesday, June 28, 1898. He was the guest of Dr. Charles E. Congdon, and made an abdominal section at the latter's private hospital during the day. In the evening Dr. Price read a paper at the Academy of Medicine entitled, Discussion and diagnosis of suppurative forms of intrapelvic disease and its surgical management. The meeting was well attended and the subject was debated by several of the members. Dr. Price returned to Philadelphia on a late train after the meeting.

---

Dr. Joseph McDowell Mathews, of Louisville, Ky., was elected president of the American Medical Association at its recent annual meeting held at Denver, Col. Dr. Mathews is one of the foremost men in the profession of medicine and the association by its choice has honored itself quite as much as it has honored him. His election is an indication that advanced methods are beginning to take hold in that body, and we predict a prosperous year in its affairs under the guidance of its new and accomplished president.

Dr. Floyd S. Crego, of Buffalo, announced, under date of June 10, 1898, that he had returned home and resumed practice. Office and residence, 469 Delaware Avenue. Hours, 8-10 a.m., 2-3 and 7-8 p. m.

Dr. Chauncey Pelton Smith, of Buffalo, announces the removal of his office from 510 Delaware avenue to No. 90 North Pearl street, corner of Allen. Hours, 8-9 a. m., 2-3 and 5.30-6.30 p.m. Telephone.

Dr. Julius Ullman, of Buffalo, has been appointed instructor in clinical medicine and assistant in the bacteriological laboratory at the University of Buffalo.

